# A Six-Year Airborne Fungal Spore Calendar for a City in the Sonoran Desert, Mexico: Implications for Human Health

**DOI:** 10.3390/jof11030183

**Published:** 2025-02-26

**Authors:** Carmen Isela Ortega-Rosas, Diana Medina-Félix, Alberto Macías-Duarte, Thanairi Gamez

**Affiliations:** 1 Cuerpo Académico de Recursos Naturales, Licenciatura en Ecología, Unidad Académica Hermosillo, Universidad Estatal de Sonora, Hermosillo 83100, Sonora, Mexico; diana.medina@ues.mx (D.M.-F.); alberto.macias@ues.mx (A.M.-D.); 2 Departamento de Geología, Universidad de Sonora, Hermosillo 83000, Sonora, Mexico; thanairig@gmail.com

**Keywords:** fungal spores, allergies, spore calendar, Sonora, climate change, human health

## Abstract

Fungal spore calendars for Mexico are non-existent. This research represents the first fungal spore concentration data in the atmosphere of Hermosillo, Mexico, a city in the Sonoran Desert with high rates of allergies and public health problems. We used standardized sampling techniques frequently used by aerobiologists, including a Burkard spore trap to monitor airborne fungal spores daily for 2016–2019 and 2022–2023. Results are expressed as daily fungal spore concentrations in air (spores/m^3^ air). The most common fungal outdoor spores corresponded to *Cladosporium* (44%), *Ascospora* (17%), Smut (14%), *Alternaria* (12%), and Diatrypaceae (7%) of the total 6-year data. High minimum temperatures produce an increase in the most important spores in the air (*Cladosporium* and *Alternaria*), whereas precipitation increases Ascospore concentrations. The most important peak of fungal spore concentration in the air is recorded during summer–fall in all cases. Airborne fungal spores at Hermosillo had a greater impact on human health. These data will be of great help for the prevention, diagnostics, and treatment of seasonal allergies in the population and for the agricultural sector that has problems with some pathogens of their crops caused by fungus.

## 1. Introduction

Aerobiological studies around the world are focused on airborne pollen concentration [1,2,3]. Few studies explore fungal spores in the air due to the difficulty of identification and the higher count effort given their extremely high concentrations compared to pollen [4]. Nevertheless, in the last two decades, the incidence of fungal diseases in humans has rapidly increased worldwide [5]. In this regard, airborne fungal spores are now considered one of the leading causes of respiratory allergies around the world as their concentration currently exceeds pollen concentration in the atmosphere by 2–3 orders of magnitude [6].

The prevalence of respiratory allergy due to fungi is not fully known; however, this allergy type is estimated to affect 20–30% of sensitive subjects (Immunoglobulin E-specific antigens (allergens) on airborne fungal spores induce type I hypersensitivity (allergic) respiratory reactions in sensitized atopic subjects, causing rhinitis and/or asthma). Fungal spores are reproductive structures produced and dispersed in large amounts and are a major contributor to the spectrum of airborne allergens [7]. Fungal sensitization is associated with increased asthma severity symptoms, morbidity, and decreased pulmonary function. Severe asthma with fungal sensitization has been described as a specific phenotype in patients with severe asthma [8,9]. Aerobiological studies estimate that 3–10% of the world’s population has some sensitivity to airborne fungal spores [10]. The World Health Organization (WHO) indicates that asthma affected 262 million people and caused 461 thousand deaths worldwide in 2019 [11].

Airborne fungal spores, commonly found in both indoor and outdoor environments, pose significant health risks to humans [3,4]. These microscopic particles, produced by various fungi such as molds, can be inhaled and trigger a range of respiratory issues, including asthma, allergic reactions, and infections—particularly in individuals with weakened immune systems. The increasing prevalence of mold in homes, workplaces, and urban environments, compounded by climate change and environmental factors, has heightened concerns about their impact on public health. As such, understanding the role of airborne fungal spores in respiratory diseases is critical for preventing long-term health consequences across diverse populations [5,6,7].

*Cladosporium* and *Alternaria* are the most common outdoor fungal spores, which show seasonal variation, peaking during the rainy seasons [1,6,7]. The outdoor concentration of fungal species from these genera has been associated with the exacerbation of asthma epidemics [5]. In Mexico, there is no published fungal–pollen calendar, although some research includes fungal spore sensitization for Mexico City [8]. For Northern Mexico, some information about airborne fungal spores has been published [1,3,12]. In Northern Mexico, the most common airborne fungal spores belong to taxa *Cladosporium*, *Alternaria*, *Aspergillus*, and Basidiospores, all positively associated to precipitation and relative humidity. For Central Mexico, a sensitization test performed on children from 2 to 18 years old [8] revealed that 7565 out of the 8794 patients displayed sensitization to ≥1 fungus taxon, including *Aspergillus*—the most common taxon—with a rate of 16.8%.

The study of fungal spore phenology has further implications for human health in subtropical arid lands, where high temperatures and high humidity—favorable conditions for fungal spread—occur during the summer. For instance, Sonoran clinical records from a public hospital at Hermosillo reported that 36 out of 279 people tested for aeroallergens tested positive for fungal spores (Basidiospores 19%; Smuts 16%; *Alternaria* 9%; Ascomycetes 9%) [1].

Airborne spores are also relevant for agriculture as they cause epiphytotic events [13]. In this regard, a fungal spore calendar is a convenient tool for both public health officials and farmers, providing summarized information on the taxonomic composition and daily concentrations of airborne fungal spores, as well as their seasonal variation. The effects of weather conditions on fungal spore concentrations are also a topic of interest. The estimation of weather effects helps in the construction of prediction models of the spore concentrations in the atmosphere [14]. Moreover, climate change will increase fungal spore production and their release into the atmosphere, accelerating the expansion of invasive plants along with their fungal parasites. This facilitated expansion may be associated with the introduction of new aeroallergens to these territories [6].

Few countries have fungal spore calendars to assess air quality [7,13,14]. We report here the six years (2016–2017–2018–2019–2022–2023) of fungal spore concentrations to build the first fungal spore calendar for an urban area in the Sonoran Desert, where the human population has high sensitization and high rates of asthma and allergy incidence due to fungal spores. This calendar will assist with the prevention, diagnostics, and treatment of both seasonal allergies in the human population and epiphytotic events in crops.

## 2. Materials and Methods

### 2.1. Study Region

**Figure 1:**
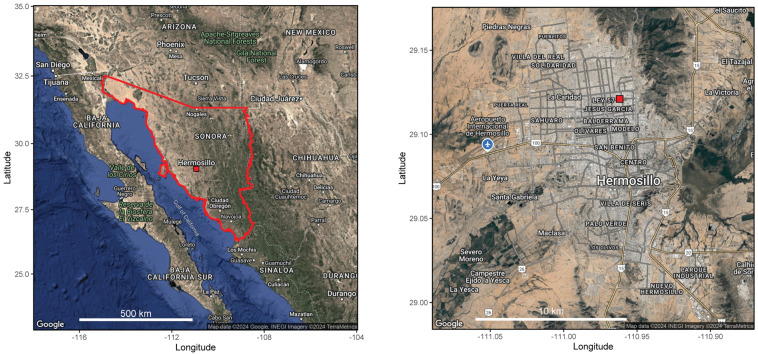
Location of airborne spore sampling site (red square) in the city of Hermosillo (**right**), state of Sonora (delimited by the red line), in NW Mexico (**left**).

This research was developed in Hermosillo, the capital city of the Mexican state of Sonora, at the center of the Sonoran Desert (29°05.02′ N, 110°57.56′ W) (Figure 1). The geomorphology of this area consists of valleys, hills, and mountains aligned in NNW-SSE direction, with elevations <700 m. The Sonoran Desert harbors high levels of biodiversity [15]. Our aerobiological monitoring station is in the northern part of the city, on the rooftop of a three-story building at Sonora State University (Figure 1). The climate in Hermosillo is a transition of two types [16]: very dry and very warm BW(h’), and very dry and semi-warm (BWh) (according to Köppen). The mean annual temperature is 25.1 °C. December is the coldest month (mean monthly temperature: 16.8 °C) and July is the warmest month (mean monthly temperature: 32.6 °C). Mean annual precipitation is 393 mm, with a summer rain regime due to the North American monsoon system. Vegetation and land-use types in the Municipality of Hermosillo include desert scrub (74.75%), agriculture (14.64%), forest (0.06%), grassland (3.03%), and others (7.52%) [16]. Hermosillo is mostly in flat terrain flanked by a mountain range to the north and east (Figure 1, left). This affects the mountain range wind direction and, consequently, the behavior of airborne particles. In this regard, there are two dominant winds: north-eastward from the Sonora coast (SW) and westward from the Sierra Madre Occidental (W). Dominant winds from SW have an average annual speed of 1.2 m s^−1^, with mean annual calms of 83%.

### 2.2. Sampling Airborne Fungal Spores

**Figure 2:**
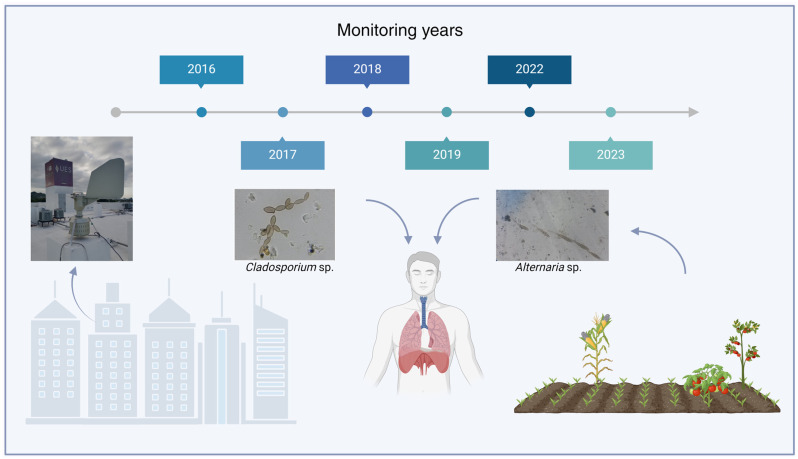
Fungal spore monitoring in Hermosillo City and potential sources of spores.

We applied a standard sampling method proposed by the Red Española de Aerobiología (Spanish Aerobiology Network, REA) [17] and used by the Red Mexicana de Aerobiología (Mexican Aerobiology Network, REMA). We sampled atmospheric spores daily from January to December over six years (2016 to 2019; 2022 to 2023), using a Hirst type of volumetric spore trap (Burkard; http://www.burkard.co.uk). This Hirst spore trap was located ~20 m above ground level on an exposed flat rooftop at Sonora State University (Figure 2). We adjusted the Hirst spore trap to aspirate 10 L air min^−1^. Fungal spores were trapped on a Melinex tape coated with an adhesive (silicone fluid). We counted fungal spores trapped on daily Melinex tape segments using an optical microscope with four longitudinal sweeps per slide and 400× magnification. Therefore, we obtained daily concentrations of spores expressed as sporesm^−^^3^ air [18]. Also, we calculated the Annual Spore Integral (ASIn), which is defined as the amount of recorded airborne fungal spores during a year [18]. Fungal spore identification was made based on morphology differentiation of spores according to several airborne spore atlases for North America [19,20].

### 2.3. Fungal Spore Calendar Construction

We summarized daily fungal spore concentrations into a fungal spore calendar. Our fungal spore calendar was created in accordance with well-established methodology [21]. Average daily fungal spore concentrations were averaged across periods of 10 days and then each 10-day period was averaged across the 6 years of our study period. Fungal spore levels are presented as averaged concentrations on a log_2_ scale. In this way, all interactions between the external factors and fungal spore concentrations were leveled out, thus enabling us to compare different fungal spore species’ concentrations throughout the year. Individual fungal spore taxa appear in the spore calendar in chronological order based on the scores of the first axis from a correspondence analysis of the data matrix, taxon vs. 10-day periods.

### 2.4. Climatic Variables

Meteorological data (mean, maximum and minimum temperatures, and total rainfall) were provided by the nearest Comisión Nacional del Agua Station to the sampling site (Figure 1). Data are shown in Supplementary Figure S1.

### 2.5. Statistical Analysis

We determined whether daily meteorological conditions influenced spore concentrations, accounting for the effects of date and annual variation. For each of the four taxa (*Cladosporium*, *Ascospora*, Smut, and *Alternaria*) that comprised >85% of the total spore concentration, we used a generalized linear model with the response variable daily spore concentration (log-transformed) with a gamma distribution and linear predictor daily minimum temperature + daily maximum temperature + daily precipitation + YEAR. Meteorological variables are intrinsically related to the date of the year. Therefore, we included a five-order sine series on the ordinal date ($\sum_{n=1}^{5}\beta_{n}\sin(2\pi n(\mathrm{date})/365)$) in the linear predictor above to unveil the effect of meteorological variables on spore concentration. We also included the factor YEAR (levels: 2016, 2017, 2018, 2019, 2022, 2023) to account for annual variation. The gamma distribution is suitable for random variables with highly skewed distribution. In this case, daily spore counts frequently show extremely high values. To account for the lagged effects of meteorological variables on spore concentrations, we also run the model described above with daily minimum temperature, daily maximum temperature, and daily precipitation lagged by 1 and/or 2 days. We evaluated the adequacy of these four models (with lags of 0 days, 1 day, 2 days, and 0 + 1 + 2 days) using Akaike’s Information Criterion (AIC) [22]. We also included a null model (without meteorological variables) in the set of models to rank by the AIC. We kept the model with the lowest AIC value (i.e., the best model) for inferences. We used Wald’s tests [23] to determine the existence of effects of the predictor variables on daily spore concentrations. We used the program R version 4.4.2 (https://www.r-project.org/, accessed on 24 January 2025) [24] for all statistical analyses and visualizations.

## 3. Results

### 3.1. Airborne Fungal Spores’ Richness of Species

Twenty-one airborne fungal spore taxa were recorded in Hermosillo during our 6-year study period (Table 1), with 14 taxa present in <1% ASIn. Fungal spore taxa with the highest concentrations were as follows: *Cladosporium* (43.70% ASIn), *Ascospora* (16.93%), Smut (14.05%), *Alternaria* (11.78%), Diatrypaceae (7.22%), Basidiospores (1.77%), and *Bipolaris* (1.29%). These taxa account for 96% in the 6 years of the Annual Spore Integral (ASIn) (Supplementary Figure S2). The average 6-year fungal spores index was ASIn = 133,218, with the highest value (ASIn = 191,448) recorded during 2016 (Table 2).

**Figure 3:**
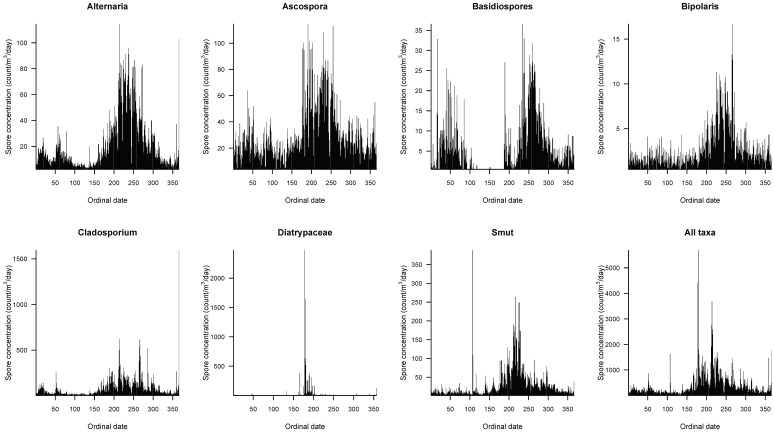
Average daily fungal spore concentration (spores/m^3^ air) in the atmosphere of Hermosillo across 2016–2029 and 2022–2023.

Fungal spore concentrations showed high seasonal variability (Figure 3). Critical spore concentrations consistently occurred during summer through the study period, with 14%, 19%, and 28% of the ASIn for June, July, and August, respectively. We also documented annual variable interannual changes in spore concentrations (Table 2): the highest monthly concentration occurred during August 2015 (33% ASIn) and August 2016 (33%), whereas the highest concentration occurred in July 2017 (22%) and in September in 2018 (20%) and 2019 (30%).

### 3.2. Fungal Spore Calendar

**Figure 4:**
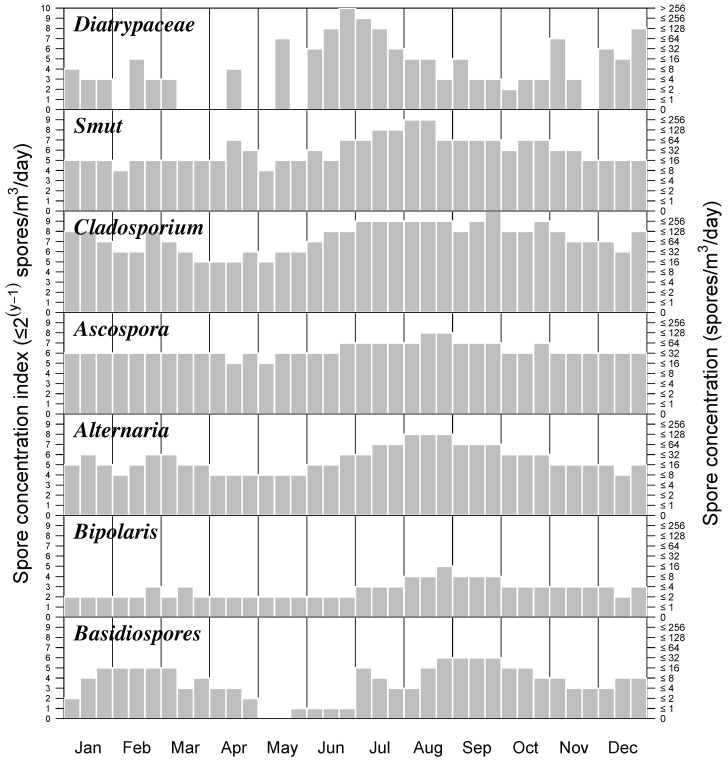
Six-year fungal spore calendar (2016–2019, 2023) in the atmosphere of Hermosillo, Mexico.

A fungal spore calendar for Hermosillo is shown in Figure 4. This represents the first atmospheric fungal spore six-year calendar not only for Hermosillo but also for Northern Mexico. Only taxa reaching a 10-day mean >1% of total concentration were included. This criterion left only seven fungal spore types on the calendar.

The species with the highest richness of fungal spores were detected between June and December and include, principally, Dyatrypaceae, Smut, *Cladosporium*, *Ascospora*, and *Alternaria*. Airborne fungal spores remain at lower concentrations during spring (Figure 4). We now describe the annual and seasonal variability in spore concentrations for each fungal taxon.

**Diatrypaceae**

Fungal spores of Diatrypaceae appear early in January and in the last two weeks of February but the highest concentrations occur in summer from June–August reaching mean daily values of 600 spores/m^3^ air during summer (Figure 3). There is a slight increase during November–December. This fungal spore is recognized as a pathogenic species for wood in angiosperms. During 2016, we recorded the highest concentrations with values doubling the six-year averages (Table 2).

**Smut**

Smut spores appear all year round, although concentrations increase during summer from June to September, reaching mean daily values of 130 spores/m^3^ air (Figure 2). This fungal spore group includes Ustilaginomycetes, Microbotryales, Urediniomycetes, and Basidiomycota, which are parasites of plants especially herbs belonging to Poaceae and Cyperaceae, both of economic importance. We recorded the highest concentrations of Smut spores in 2016, with values doubling the 6-year average (Table 2).

***Cladosporium* sp.**

*Cladosporium* fungal spores are one of the most abundant airborne allergens worldwide. This taxon is present all year round in Hermosillo, at high concentrations too, peaking from June to October (Figure 2) and reaching mean daily concentrations of 300 spores/m^3^ air. During 2016 and 2023, we recorded the highest concentrations with values considerably higher than the 6-year average (Table 2).

***Ascospora***

*Ascospora* spores are present year-round at low concentrations. The highest peak concentration also occurs in summer, from June–September, reaching mean daily values around 60 spores/m^3^ air (Figure 3). During 2023, we recorded the highest concentrations with values three times higher than the 6-year average (Table 2).

***Alternaria* sp.**

*Alternaria*, which commonly grows as a parasite on vegetation, is the major environmental allergen associated with asthma worldwide. *Alternaria* is present all year in Hermosillo. The period with the highest concentrations peaks also occurs in the summer from June–October, reaching mean daily values around 60 spores/m^3^ air (Figure 3). During 2016, we recorded the highest concentration values, 1.5 times higher than the 6-year average (Table 2).

***Bipolaris* sp.**

*Bipolaris* spores are present all year round at low concentrations (<4 spores/m^3^ air), although its concentration increases during summer from June–September, with mean daily concentrations of 8 spores/m^3^ air (Figure 3). During 2023, we recorded the highest concentrations with values 2 times higher than the 6-year average (Table 2).

**Basidiospores**

Fungal spores of Basidiospores are present during two periods of the year from January–April and from July–December. The highest concentrations were recorded during summer–fall from June–October, reaching mean daily concentrations of 15 spores/m^3^ air (Figure 3). During 2023, we recorded the highest concentrations with values 3 times higher than the 6-year average (Table 2).

### 3.3. Climate and Spores’ Concentrations in Air

We found strong evidence that daily spore concentration is related to daily meteorological conditions after accounting for seasonal and annual variations. We also found lagged effects of meteorological variables on spore concentrations (Table 3). High daily minimum temperatures promoted higher spore concentrations. Daily minimum temperature had a strong increasing effect on daily spore concentrations for *Cladosporium* and *Alternaria.* This strong increasing effect of daily minimum temperature on spore concentration lagged two days for *Cladosporium* (Table 3).

High maximum temperatures inhibited spore production. Daily maximum temperature had a strong decreasing effect on daily spore concentration for *Cladosporium.* This strong decreasing effect of daily minimum temperature on spore concentrations lagged 2 days for *Alternaria*, *Ascospora*, and Smut (Table 3).

Finally, high daily precipitation promoted spore production only for *Ascospora* and showed no effect for *Alternaria*, *Cladosporium*, and Smut. Daily precipitation from both the current day and the day before had a strong increasing effect on current daily spore concentration for *Cladosporium* (Table 3).

## 4. Discussion

A fungal spore calendar is a powerful tool for agricultural, public health, and aerobiological research. In agriculture, the monitoring of fungal spores is highly relevant to the study of the life cycles of parasites and to developing plant protection plans. For human health, the detection of above-threshold airborne concentration events for allergenic taxa (e.g., *Alternaria* and *Cladosporium*) helps in the diagnosis and treatment of inhaled allergens [25].

Allergenic fungal spores recorded in Hermosillo are usually present throughout the year (Figure 3 and Figure 4) but high concentrations occur at the onset of the summer and fall seasons (Figure 3). The most important airborne fungal spores in the six-year average were *Cladosporium*, *Ascospora*, Smut, and *Alternaria.* This composition correlates well with numerous studies that report that *Cladosporium* and *Alternaria* are the most dominant spore types at most monitoring stations around the world [5,7,8,14,26]. As in other studies of fungal spores in Sonora [12], *Alternaria* and *Cladosporium* together with *Aspergillus* were the dominant taxa during 2011 at Ciudad Obregon in southern Sonora. Our ASIn indicates a high interannual variation in fungal spores in air during the years of monitoring. The 6-year average ASIn was 133,218, which exceeds those reported for Obregon in 2008 (917 spores) and 2011 (1690 spores) [12]. An ASIn of 11,000 has been reported for the tropics [27], whereas an ASIn of 3500–54,595 has been reported for dry climates in urban environments [28], both much lower than ours. We recorded the lowest ASIn (68,130 spores/m^3^) air during 2019 and the highest ASIn in 2016 (191,448) and 2023 (189,600). Those values are higher than those reported in other cities of Mexico but are lower than those of other cities in Europe such as Szczecin, Poland (595,199) [14], and Bratislava, Slovakia (83,641) [7]. In North America, several studies found higher concentrations of fungal spores in desert cities including Las Vegas (USA) [26]. Although ASIn is not reported for Las Vegas, the total fungal spore concentration in air was 6393 spores/m^3^ in May of 2015, comparable to the concentration that we recorded in Hermosillo during the highest peak in May of 2023 with 8445 spores/m^3^. Another study in New Orleans (USA) showed lower values of fungal spore concentrations than those recorded in Hermosillo, ranging from 33,179 to 66,167 spores/m^3^ [29].

Sonora not only has a higher rate of ASIn than those documented for regions in Mexico but also we document an increase in the total annual amount of spores in the air, showing an important interannual variation (Table 2). In 2016 and 2023, we recorded the highest ASIn with 191,448 and 189,600 spores/m^3^ air. These peaks coincidentally occurred during El Niño years [30]. The El Niño causes above-average winter precipitation in Sonora. As suggested by our statistical analysis, Ascospores are positively influenced by precipitation and this could explain the higher concentrations reached during 2016 (ASIn = 15,837 spores/m^3^ air) and 2023 (ASIn = 68,241 spores/m^3^ air). Both genera were present with higher concentrations in those years (Table 2). Further research about the influence of ENSO in airborne fungal spores is mandatory in light of this research.

Regarding the most important airborne fungal spores that we recorded, *Cladosporium* spores are present year-round but spore concentrations increase from June to October; this correlates well with the previous studies at Sonora [1] and with a fungal record from a similar desert city in the United States [26], where *Cladosporium* was present through the summer and fall months. High daily minimum temperature increases may favor the production and release of *Cladosporium* spores in the air, whereas the daily maximum temperature inhibits the spore concentrations at 0 and 2 delay days (Table 3).

*Ascospore* spores, the second spore group with high concentrations in the air, were present more consistently all year but concentrations increased from June to September in summer months related to the monsoon season, supporting the findings of previous work in Hermosillo [1]. Our statistical analysis shows that these fungal spores are positively correlated with precipitation at 0- and 2-day lags (Table 3).

Smut spores were the third spore group with high concentrations in the air, present in lowest concentrations through spring but increasing considerably during summer–fall, as previously reported [1]. Minimum temperature increases also trigger an increase in Smut spores in the air (Table 3) with 0- and 2-day lags. Also, the daily maximum temperature inhibits spore concentrations with a 2-day lag.

*Alternaria* spores were the fourth-dominant spore in the air, being present all year but whose concentrations increase from June to November. These results are similar to those documented by previous research in Sonora [1] and at another desert city in the United States [26]. A daily minimum temperature increase favors the production and release of these spores in air with 0- and 2-day lags. In contrast, daily maximum temperature inhibits spore concentrations with a 2-day lag (Table 3).

**Figure 5:**
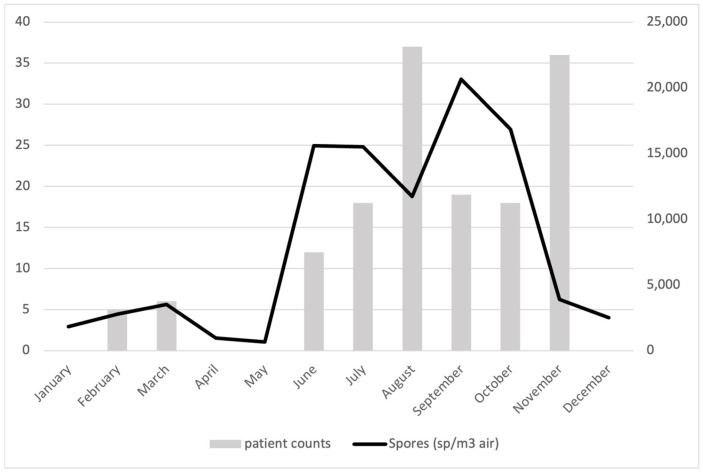
Monthly count of patient’s positive sensitization to fungal spores at a public hospital (left axis) and airborne fungal spore concentration in Hermosillo during 2018 (right axis).

*Alternaria* and *Cladosporium* genera are the most important outdoor allergens [25,31]. Sensitization and exposure to those spore types are related to the development of asthma and rhinitis. Epidemics of asthma may exacerbate into life-threatening forms of asthma [5]. In this regard, the Sonora State Health Secretary reports that 13,454 people in Hermosillo presented diseases related to allergies in 2016 [1]. This agency also reports that the highest number of patients attended occurred during summer and fall, associated with the highest peaks of fungal spores in the atmosphere (Figure 3 and Figure 4). A study of sensitization to fungal spores from skin tests—taken between 2004 and 2015 in patients between 2 and 18 years old in Mexico City [8] who presented some type of allergic condition—indicated that 7565 out of the 8794 patients displayed sensitization to at least one fungus. A remarkable prevalence was observed for *Alternaria* (36%), followed by *Aspergillus* (27%), *Cladosporium* (18%), and Penicillium (13%) [8]. Data provided by the Hospital General Zona Sur del IMSS in Hermosillo for 2018 indicate that 90% of patients arriving with allergy symptoms (151 people for 2018) tested positive for allergy to fungal spores. The most important fungal spores were Zygomycetes (23%), *Alternaria* (21%), Ascomycetes (20%), *Cladosporium* (19%), and Smuts (18%). Overall, the period when people presented more symptoms correlates well with the higher concentrations of fungal spores in the air recorded in this work (Figure 5) from June to November (in summer–fall).

## 5. Conclusions

Extremely high concentrations of airborne fungal spores are recorded at the desert city of Hermosillo, Sonora, which requires explicit attention from public health agencies. The most important taxa are *Cladosporium*, *Alternaria*, Ascospores, and Smuts. Fungal spore concentrations show high annual and interannual variability. The period with the highest peak in airborne fungal spore concentrations is centered around the summer–fall, after the monsoon season. Our fungal spore calendar is a first for Mexico, which will be of great application for public health. Fungal spores in the air can trigger adverse effects on the human immune system and lead to respiratory diseases, such as asthma and allergies. This calendar will also find applications in agriculture to control fungi that can cause epiphytotic events in crops. For grape—which is a major crop in the municipality of Hermosillo—*Cladosporium* sp. produces berry rot, while *Alternaria* sp. causes spots on the leaves of grape crops. In both cases, these fungi produce a loss in grape (*Vitis vinifera*) production in the region.

## Figures and Tables

**Table 1:** Mean airborne fungal spore types collected in Hermosillo, Sonora, over 6 years. Average percentages for the study period are reported.

Taxa	Total Spores	Percentage (%)
*Cladosporium*	298,357	43.70
*Ascospora*	115,629	16.93
Smut	95,903	14.05
*Alternaria*	80,451	11.78
Diatrypaceae	49,279	7.22
Basidiospores	12,093	1.77
*Bipolaris*	8820	1.29
Myxomicetes	5639	0.83
Pithomyces	5046	0.74
*Agaricus*	4954	0.73
*Arthrinium*	2562	0.38
*Curvularia*	1856	0.27
*Torula*	862	0.13
*Periconia*	500	0.07
*Sporidesmium*	310	0.05
*Boerlagella*	291	0.04
*Spegazzinia*	117	0.02
*Leptosphaeria*	116	0.02
*Peronospora*	6	0.00
*Beltrania*	1	0.00
*Fuligo*	1	0.00
ASIn	682,793 ^1^	100%

^1^ Annual Spore Integral (ASIn) for the total years of monitoring.

**Table 2:** Fungal spore total year concentrations (spores/m^3^ air) of most important taxa for the study period. Fungal Annual Spore Integral (ASIn) is also reported.

Taxa	2016	2017	2018	2019	2023	Average
*Cladosporium*	73,205	44,876	55,439	36,455	80,510 ^1^	58,097
*Ascospora*	15,837	13,265	7520	5633	68,241	22,099
Smut	50,601	24,662	10,661	9161	0	19,017
*Alternaria*	20,252	18,279	12,905	9716	17,487	15,728
Diatrypaceae	22,021	11,990	5183	2758	7296	9850
Basidiospores	96	2410	1583	1439	6121	2330
*Bipolaris*	1249	856	1367	1304	3663	1688
Myxomicetes	2481	1420	854	764	0	1104
*Pithomyces*	322	115	104	73	4431	1009
Agaricus	3817	1133	0	0	0	990
*Arthrinium*	855	845	409	414	0	505
*Curvularia*	1	0	0	0	1851	370
*Torula*	356	224	169	101	0	170
*Periconia*	8	102	186	186	0	96
*Sporidesmium*	93	86	69	56	0	61
*Boerlagella*	116	79	48	45	0	58
*Spegazzinia*	21	47	27	19	0	23
*Leptosphaeria*	116	0	0	0	0	23
*Peronospora*	0	0	0	6	0	1
*Beltrania*	0	1	0	0	0	0
*Fuligo*	1	0	0	0	0	0
ASIn	191,448	120,390	96,524	68,130	189,600	133,218

^1^ *Cladoporium* has the highest concentration in air in 2023 compared to the entire monitoring period.

**Table 3:** Effects of meteorological variables on spore concentration in Hermosillo, Mexico. Symbols + and − denote gamma regression coefficients β>0 and β<0, respectively. Single, double, and triple symbols denote *p* values <0.01, <0.01, and <0.001 for Wald’s test (β=0). Symbol 0 (no effect) denotes gamma regression coefficients of Wald’s test with *p* values > 0.05. All models include an intercept term, variable date and factor YEAR (levels: 2015, 2016, …, 2023), all omitted in this table. Gamma regression model for each taxon was the best of five models (see text) by means of Akaike’s Information Criterion.

Parameter (Lag in Days)	*Cladosporium*	*Ascospora*	Smut	*Alternaria*
Min. Temp. (0)	+ + +	+	+ + +	+ + +
Min. Temp. (1)	0	+	0	0
Min. Temp. (2)	+ +	+ + +	+ +	+ +
Max. Temp. (0)	− − −	0	0	0
Max. Temp. (1)	0	0	0	0
Max. Temp. (2) ^1^	− −	− − −	− − −	− − −
Precipitation (0)	0	+ + +	0	0
Precipitation (1)	0	+ + +	0	0
Precipitation (2)	0	0	0	0

^1^ The daily maximum temperature inhibits spore concentration in all cases after 2 days.

## Data Availability

Data supporting the reported results can be found on demand at the official page of the Mexican Aerobiology Network (http://rema.atmosfera.unam.mx/rema/Default.aspx, accessed on 15 December 2024) or upon request from the corresponding author.

## Supplementary Materials

The following supporting information can be downloaded at https://www.mdpi.com/article/10.3390/jof11030183/s1, Figure S1: Images of most common fungal spores under microscopy; Figure S2: Meteorological variables during the study period.
