# Supplementary material for: A Six-Year Airborne Fungal Spore Calendar for a City in the Sonoran Desert, Mexico: Implications for Human Health
Source: J Fungi (Basel). 2025 Feb 26;11(3):183. doi: 10.3390/jof11030183 (PMC11943160; doi:10.3390/jof11030183)
Supplement: Supplementary file 1 [file jof-11-00183-s001.zip › jof-3439746-supplementary.pdf]

Supplementary Figure 1

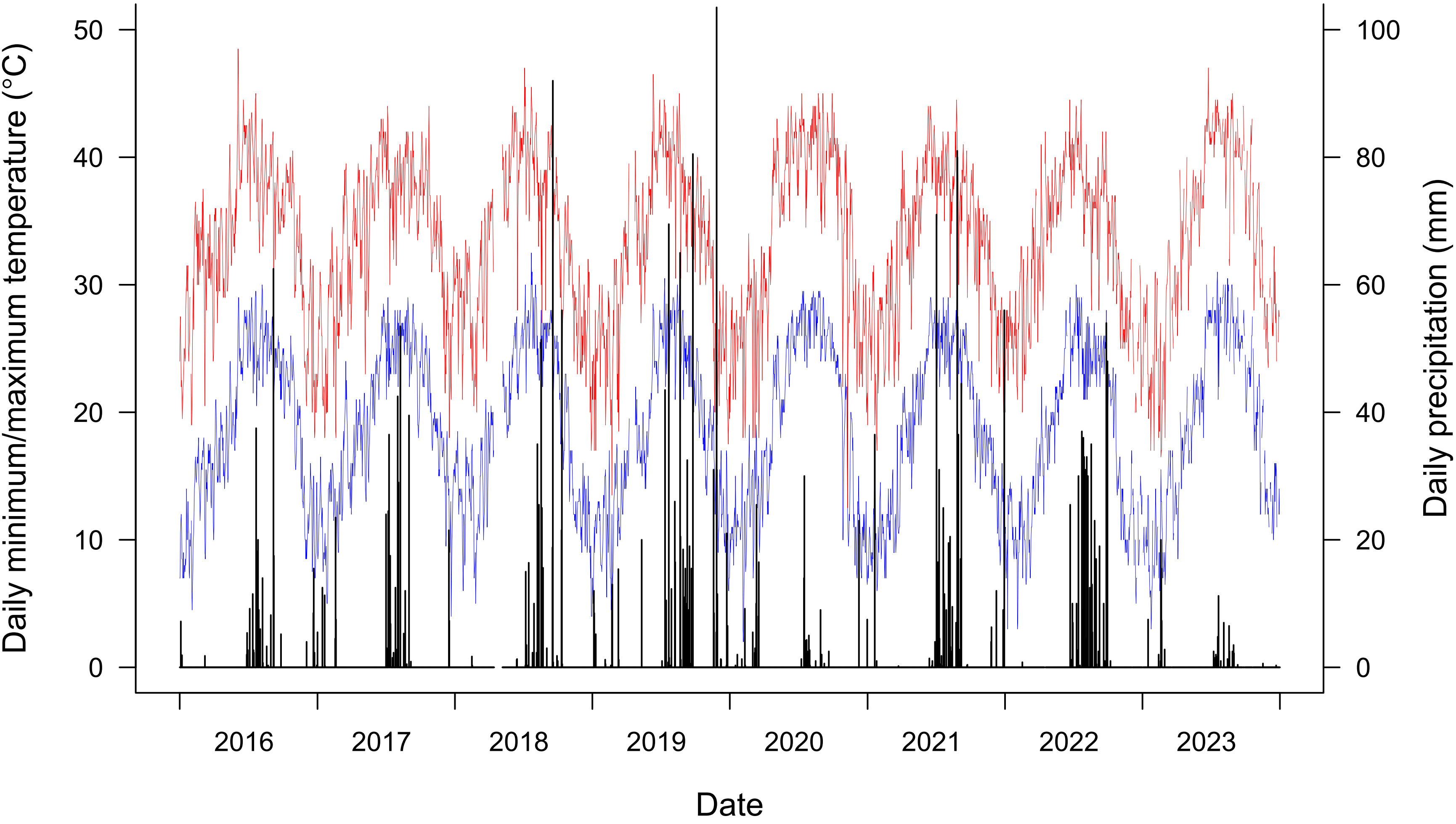

Supplementary Figure 2

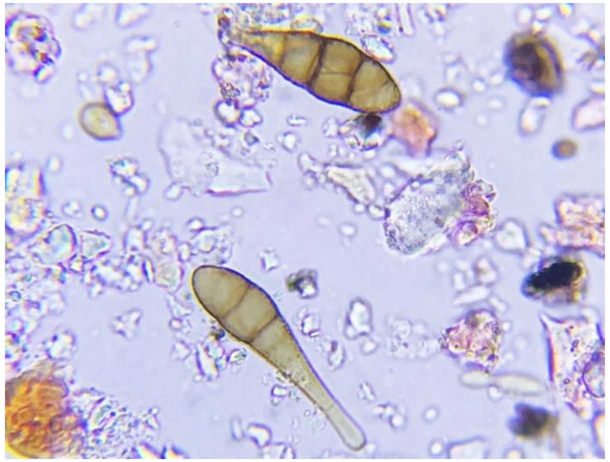

Alternaria Sp. (100x) ~40μ

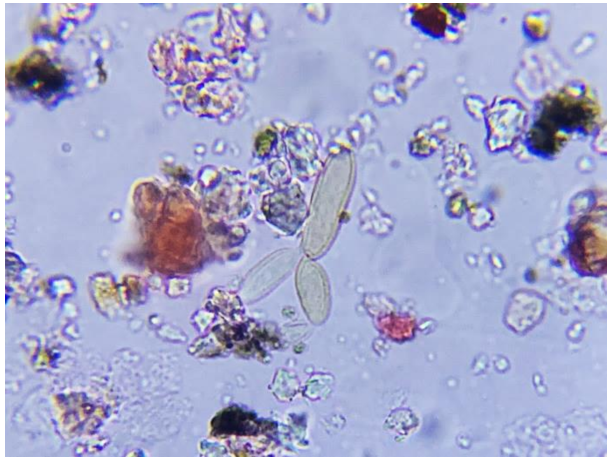

Cladosporium Sp. (100x) ~10μ

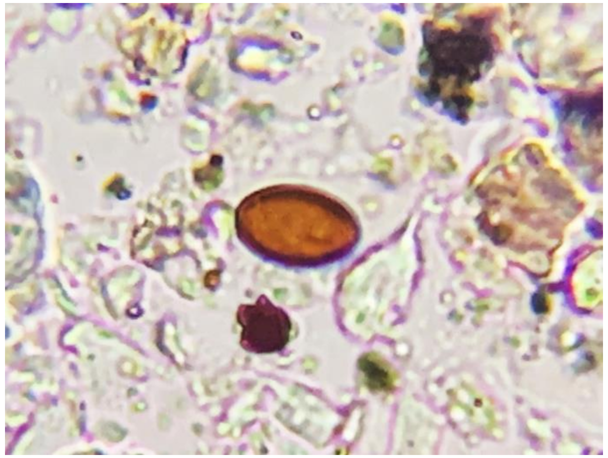

Ascospora (100x) ~30μ

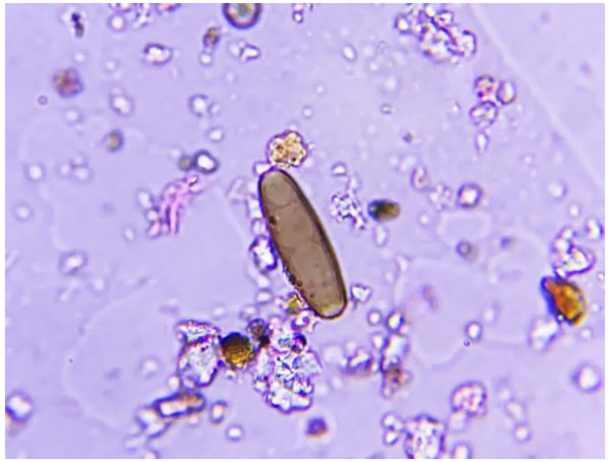

Bipolaris Sp. (100x) ~30μ

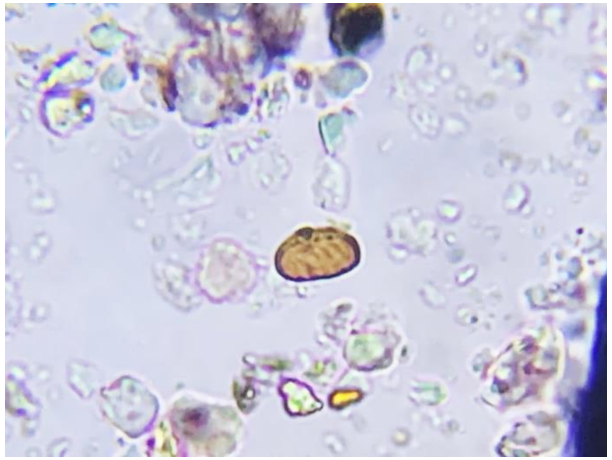

Basidiospora (100x) ~10μ

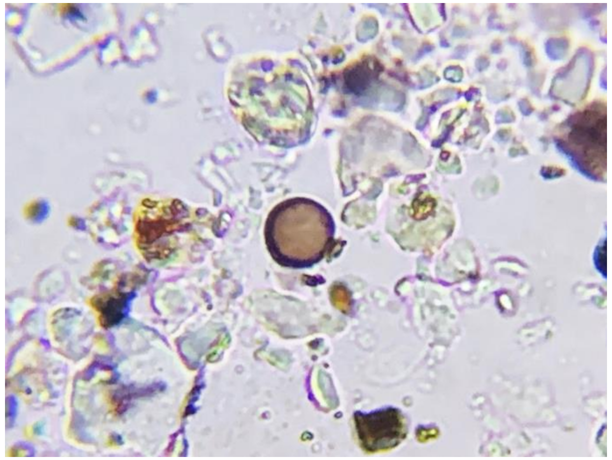

Smut (100x) ~20μ
